# Transporter-Mediated Drug Interaction Strategy for 5-Aminolevulinic Acid (ALA)-Based Photodynamic Diagnosis of Malignant Brain Tumor: Molecular Design of ABCG2 Inhibitors

**DOI:** 10.3390/pharmaceutics3030615

**Published:** 2011-09-14

**Authors:** Toshihisa Ishikawa, Kenkichi Takahashi, Naokado Ikeda, Yoshinaga Kajimoto, Yuichiro Hagiya, Shun-ichiro Ogura, Shin-ichi Miyatake, Toshihiko Kuroiwa

**Affiliations:** 1 Omics Science Center, RIKEN Yokohama Institute, 1-7-22 Suehiro-cho, Tsurumi-ku, Yokohama,230-0045, Japan; 2 Department of Neurosurgery, Osaka Medical College, Osaka 569-8686, Japan; 3 Graduate School of Bioscience and Biotechnology, Tokyo Institute of Technology, Yokohama 226-8501, Japan

**Keywords:** brain tumor, glioma, photodynamic diagnosis, 5-aminolevulinic acid (ALA), ABC transporter, ABCG2, protein kinase inhibitor

## Abstract

Photodynamic diagnosis (PDD) is a practical tool currently used in surgical operation of aggressive brain tumors, such as glioblastoma. PDD is achieved by a photon-induced physicochemical reaction which is induced by excitation of protoporphyrin IX (PpIX) exposed to light. Fluorescence-guided gross-total resection has recently been developed in PDD, where 5-aminolevulinic acid (ALA) or its ester is administered as the precursor of PpIX. ALA induces the accumulation of PpIX, a natural photo-sensitizer, in cancer cells. Recent studies provide evidence that adenosine triphosphate (ATP)-binding cassette (ABC) transporter ABCG2 plays a pivotal role in regulating the cellular accumulation of porphyrins in cancer cells and thereby affects the efficacy of PDD. Protein kinase inhibitors are suggested to potentially enhance the PDD efficacy by blocking ABCG2-mediated porphyrin efflux from cancer cells. It is of great interest to develop potent ABCG2-inhibitors that can be applied to PDD for brain tumor therapy. This review article addresses a pivotal role of human ABC transporter ABCG2 in PDD as well as a new approach of quantitative structure-activity relationship (QSAR) analysis to design potent ABCG2-inhibitors.

## 1. Introduction

In the 1960s Lipson and Baldes introduced a hematoporphyrin derivative (HpD), a product derived following treatment of hematoporphyrin with a mixture of acetic and sulfuric acids and sodium hydroxide [1]. Their development of the hematoporphyrin derivative established the basis of today's photodynamic diagnosis (PDD) and photodynamic therapy (PDT) [2-8]. PDD and PDT are achieved by a photon-induced physicochemical reaction which is induced by excitation of photosensitizer exposed to light.

In recent years, remarkable advances were made in PDD technology that makes it easier to reliably achieve complete excision of malignant gliomas [9-11] and meningiomas [12]. While the extent of tumor resection that should be undertaken in patients with glioblastoma multiforme remained controversial [13,14], fluorescence-guided gross-total resection has been developed and it has prolonged the survival time of glioblastoma and meningioma patients [9-12,15,16]. Historically, two fluorescent agents, *i.e.*, fluorescein sodium and protoporphyrin IX (PpIX) induced by 5-aminolevulinic acid (ALA) or its ester, have been used in glioma surgery. Because of its high tumor specificity and safety, ALA is particularly promising. It actively accumulates in the neoplasm and is converted to PpIX which is fluorescent. This phenomenon has been clinically applied to the detection of neoplasms in the brain and other organs, such as the bladder, skin, and bronchus. Fluorescence-guided resection is considered beneficial for the removal of complicated or malignant tumors that have a high risk of recurrence.

## 2. Biosynthesis and Metabolism of Porphyrins and Heme

**Figure 1.**
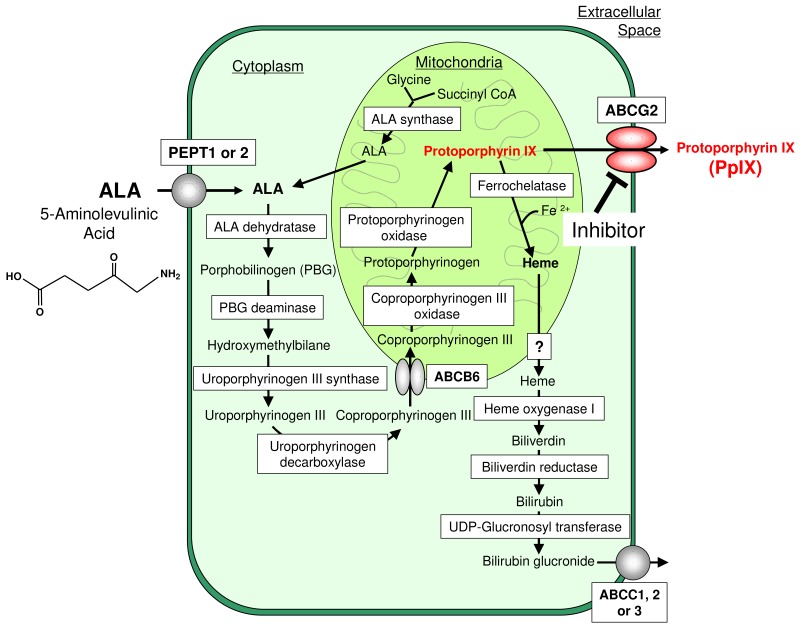
Schematic illustration of the biosynthesis and catabolism of porphyrins and heme. Rectangles indicate the enzymes involved in the metabolism reactions. Porphyrins are synthesized from glycine and succinyl CoA via eight-stepped enzymatic reactions. ALA, γ-aminolevulinic acid; PBG, porphobilinogen. Heme is formed from protoporphyrib IX (PpIX) by the action of ferrochelatase. Heme, thus formed, is catabolized to biliverdin by the microsomal enzyme heme oxygenase 1. Biliverdin is subsequently metabolized to bilirubin by biliverdin reductase. ABC transporters ABCC1, ABCC2, and ABCC3 play a role of eliminating bilirubin-glucuronide conjugate from cells. UDP, uridine diphosphate. ABC transporter ABCB6 is considered to be responsible for the import of coproporphyrinogen III into mitochondria, whereas ABCG2 transports porphyrins across the plasma membrane to maintain intracellular porphyrin homeostasis. ABCG2-mediated porphyrin transport can be inhibited by drugs, such as gefitinib and protein kinase inhibitors.

In our body, porphyrins and heme play critical roles in diverse biological processes, such as respiration and oxidative metabolism [17,18]. Heme is synthesized via an eight-stepped pathway that is spatially shared between mitochondria and cytoplasmic compartments (Figure 1). Both biosynthesis and its intracellular concentration of cellular porphyrins and heme are tightly regulated. At the first step, ALA is synthesized from glycine and succinyl Co-A in a reaction catalyzed by ALA-synthase and regulated by the intracellular free heme pool. After ALA synthesis, a sequence of reactions occur leading to the production of various porphyrin compounds. Finally, as a result of ferrochelatase action, ferrous iron is incorporated into PpIX to form heme. Heme production, as well as the synthesis of its two immediate precursors (PpIX and protoporphyrinogen) occurs in the mitochondria. ABCB6, one of human ABC transporters, reportedly transports coproporphyrinogen III from the cytoplasm to the mitochondria [19], whereas another ABC transporter ABCG2 is responsible for the cellular homeostasis of porphyrins and their related compounds [20]. Disturbances in cellular porphyrin biosynthesis or metabolism are associated with several types of porphyria, which represent an elevation of photo-toxic hemeprecursors including PpIX [21-23].

## 3. Enforced Biosynthesis of Protoporphyrin IX in Cancer Cells by ALA Administration

Exogenous ALA administration short circuits the first step of porphyrin biosynthesis (Figure 1), where ALA is transported into cancer and normal cells by oligopeptide transporter 1 or 2 (PEPT1 or PEPT2) [24-27]. ALA induces the accumulation of detectable amounts of PpIX in certain types of cells, including cancer cells, making them photosensitive [28]. ALA-induced endogenous PpIX accumulation thus constitutes a photosentization process in which the selectivity of neoplastic cells in synthesizing and/or accumulating PpIX may be exploited to enhance the efficacy of PDT and PDD.

ALA-induced PpIX biosynthesis has many other advantages. PpIX is an essentially monomeric compound with a high fluorescence yield [29] and photosensitizing capability due to its good singlet oxygen quantum efficiency [30,31], and is rapidly metabolized *in vivo* [32]. Animal and human studies have shown that ALA induces PpIX clearance from the skin within 24 h after systemic, topical or intradermal administration [28], whereas hematoporphyrin derivatives cause prolonged skin photosensitivity (1 to 2 months). In this context, ALA administration is advantageous with respect to the drug safety.

## 4. Photodynamic Diagnosis and Fluorescence-Guided Microsurgery

**Figure 2.**
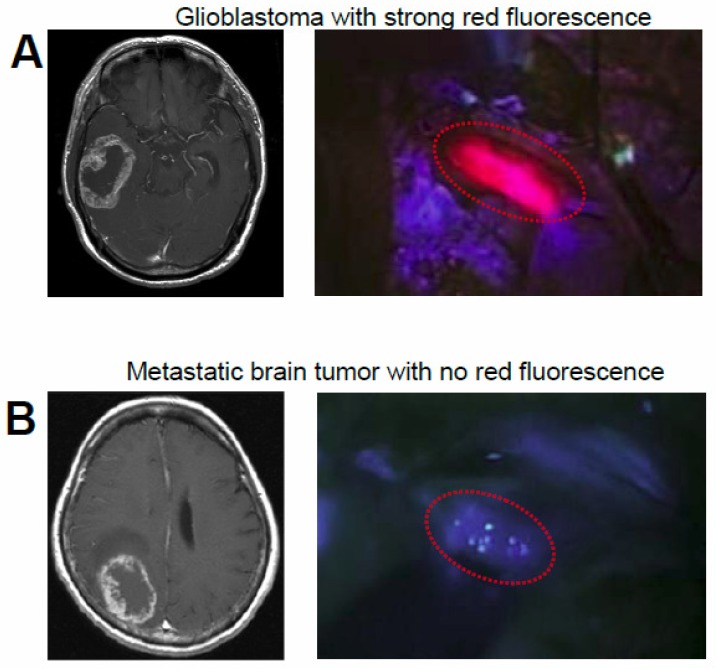
Preoperative Gd-enhanced MRI scanning and 5-ALA-induced tumor fluorescence. (**A**) Glioblastoma with strong red fluorescence; (**B**) metastatic brain tumor with no red fluorescence. Circular lines show the tumor location in operative view. Data are from reference [33].

In PDD and fluorescence-guided neurosurgery [9,10,12,15,16], ALA is used for intra-operative labeling of the border regions of malignant gliomas infiltrated by alive clonogenic tumor cells and is helpful in precise resection of those regions. ALA is converted to PpIX in living cells and emits strong red fluorescence, with the excitation of blue-violet light. As PpIX preferentially accumulates in the tumor tissue in comparison with normal tissue, this red fluorescence becomes a good hallmark for discrimination between normal and tumor tissues, especially in malignant gliomas, which have infiltrative characteristics. Approximately 80% to 90% of the malignant gliomas show this red fluorescence in surgery (Figure 2A), while only a limited number of metastatic brain tumor cases do not (Figure 2B). In the surgery for metastatic brain tumor and lesionectomy for radiation necrosis and neurodegenerative disease, white matter around the lesion shows weak and vague fluorescence; this also provides us with a hallmark in the surgery. Additionally, in meningioma, some tumors showed the red fluorescence, which is especially helpful in the removal of the infiltrative portion in the bone and normal parenchyma [12]. Clinical data indicate that ALA-photodynamic diagnosis-assisted resection of malignant gliomas results in statistically significant prolongation of postoperative survival [15,16]. Ongoing research concentrates also on the use of ALA for a selective elimination of glioma cells *in situ*, and on lipophilic ALA derivatives with more favorable pharmacokinetic properties.

There is a question still remaining unanswered, namely: Why does PpIX accumulate in the tumor tissue more preferentially than in normal tissue? Until recently, little has been known about the molecular mechanisms underlying PpIX accumulation in clinical malignant brain tumors following 5-ALA administration. We have hypothesized that malignant brain tumors might exhibit distinct gene expression patterns associated with activated enzymes or transporters in the porphyrin-biosynthesis pathway and that such differences in genetic expression patterns would represent the fluorescence intensity in malignant brain tumors.

**Figure 3.**
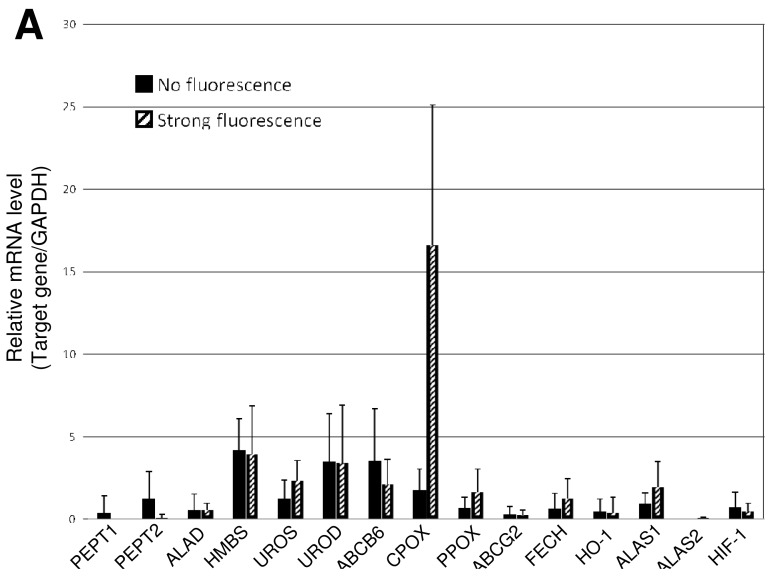

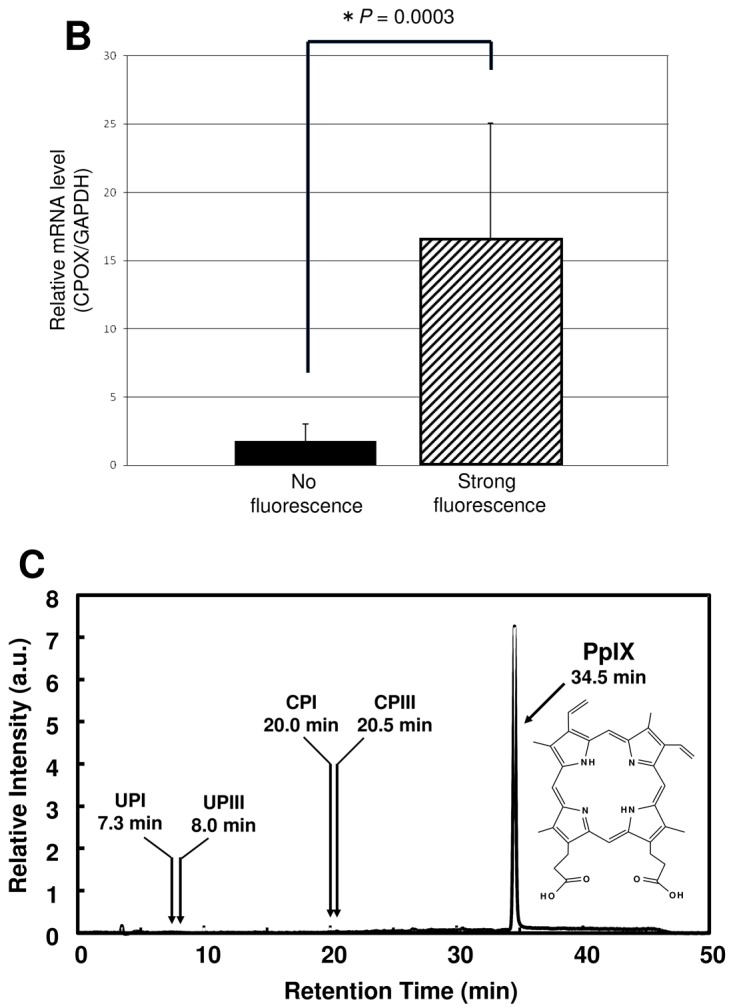
Enhanced expression of *CPOX* gene in glioblastoma with strong red fluorescence. (**A**) Comparison of the mRNA levels of *PEPT1*, *PEPT2*, *ALAD*, *HMBS*, *UROS*, *UROD*, *ABCB6*, *CPOX*, *PPOX*, *ABCG2*, *FECH*, *HO-1*, *ALAS1*, *ALAS2*, and *HIF-1* genes between the “no fluorescence” and “strong fluorescence” groups. Gene names: *PEPT1*, oligopeptide transporter 1; *PEPT2*, oligopeptide transporter 2; *ALAD*, delta-aminolevulinate dehydratase; *HMBS*, hydroxymethylbilane synthase; *UROS*, uroporphyrinogen III synthase; *UROD*, uroporphyrinogen decarboxylase; *ABCB6*, ABC transporter B6; *CPOX*, coproporphyrinogen oxidase; *PPOX*, protoporphyrinogen oxidase; *ABCG2*, ABC transporter G2 (BCRP); *FECH*, ferrochelatase; *HO-1*, heme oxygenase 1; *ALAS1*, 5-aminolevulinate synthase 1; *ALAS2*, 5-aminolevulinate synthase 2; *HIF-1*, hypoxia inducible factor-1 alpha subunit. Total RNA was extracted from brain tumor areas, and the first strand cDNA was prepared from the extracted total RNA in a reverse transcriptase (RT) reaction. Quantitative PCR was performed as described previously [33]. The mRNA levels of these genes are normalized to the mRNA level of glyceraldehyde-3-phosphate dehydrogenase (*GAPDH*). Data are expressed as means ± SD (*N* = 10) [33]; (**B**) Statistical analysis of *CPOX* mRNA levels between the “no fluorescence” and the “strong fluorescence” groups. Data are expressed as means ± SD (*N* = 10) [33]. (**C**) HPLC elution profile of porphyrins in brain tumor samples with strong red fluorescence. HPLC analysis was performed as described previously [34]. As the standards, uroporphyrin 1 (UPI), Uroporphyrin III (UPIII), Coproprophyrin I (CPI), Coproprophyrin III (CPIII), and protoporphyrin IX (PpIX) were eluted at retention times of 7.3, 8.0, 20.0, 205, and 34.5 min, respectively.

In order to answer the question and to examine our hypothesis, we analyzed the expression levels of major enzymes and transporters involved in biosynthesis and metabolism of porphyrin. By quantitative real-time PCR (qRT-PCR), we measured the mRNA levels of key genes in a total of 20 tumor samples that had been surgically resected from brain tumor patients [33]. We found that the level of mRNA encoding coproporphyrinogen oxidase (CPOX) was remarkably increased in malignant brain tumors that exhibited strong fluorescence of PpIX after 5-ALA administration (Figure 3A). The high mRNA level of CPOX expression was significantly well correlated with the phenotype of strong 5-ALA induced fluorescence (Figure 3B) and accumulation of PpIX in malignant brain tumors (Figure 3C). These findings were further confirmed by immunohistochemical studies with a CPOX-specific antibody, as well [33]. Thus, it has been concluded that the elevated levels of CPOX protein as well as its mRNA are one of the major mechanisms underlying the ALA-induced PpPIX fluorescence in malignant brain tumors.

Gupta *et al.*, on the other hand, reported that ABCG2 mRNA was present in normal colorectal tissue, but showed a 6-fold decrease in colorectal cancer [35]. The down-regulation of ABCG2 mRNA and protein was evident in cervical cancer as well [35]. In addition, we have also found that ABCG2 expression was down-regulated to some extents in malignant glioma of human brain tumors. The mRNA level of ABC transporter ABCG2 was lower in malignant glioma cells in the brain tumor that exhibited strong fluorescence of PpIX after ALA treatment, whereas the surrounding cells emitted weak and vague fluorescence. Since ABCG2 is responsible for elimination of porphyrins from living cells, reduced expression of ABCG2 can facilitate the accumulation of PpIX in malignant glioma cells. Taken together, it is strongly suggested that both up-regulation of CPOX and down-regulation of ABCG2 are important factors involved in the high accumulation of PpIX in certain cancer cells, including malignant glioma cells.

## 5. Human ABC Transporter ABCG2 in Cancer Chemotherapy and PDT

**Figure 4.**
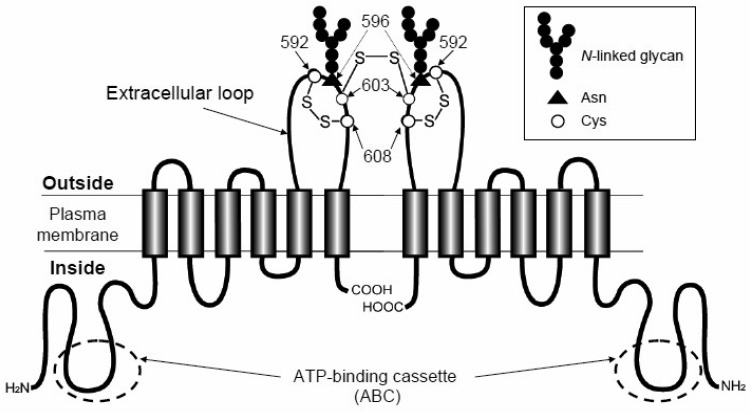
Schematic illustrations of ABCG2 protein structure. The ABCG2 protein expressed in the plasma membrane is a homodimer linked via a cysteinyl disulfide bond. The cysteine residue corresponding to Cys603 of human ABCG2 is involved in homodimer formation. The substrate-binding site is formed when ATP is bound to the ATP-binding cassettes (ABC) of the ABCG2 homodimer. Disulfide bond formation at Cys603 does not appear to be prerequisite for exerting the transport activity of ABCG2.

Human ABC transporter ABCG2, originally named Breast Cancer Resistance Protein (BCRP), was first discovered in doxorubicin-resistant breast cancer cells [36]. Since the same transporter has also been found in the human placenta [37] as well as in drug-resistant cancer cells selected in mitoxantrone [38], the transporter was also called ABCP or MXR1. The *ABCG2* gene is located on chromosome 4q22 and spans over 66 kb, comprising 16 exons and 15 introns. ABCG2 is classified in the G-subfamily of human ABC transporter genes according to the designated international nomenclature. Compared with the molecular structures of the well-known multidrug resistance transporters ABCB1 (P-gp/MDR1) and ABCC1 (MRP1), ABCG2 is a so-called “half ABC transporter” bearing six transmembrane domains and one ATP-binding cassette. Human ABCG2 has recently been shown to exist in the plasma membrane as a homodimer bound through disulfide-bonded cysteine residues [16,38,40] (Figure 4). Treatment with mercaptoethanol reduced the apparent molecular weight of ABCG2 from 140,000 to 70,000. Based on the cDNA sequence, a total of eleven cysteine residues exist in the ABCG2 protein. Among them, three cysteine residues in the extra-cellular loop of ABCG2 play pivotal roles in homodimer formation or protein expression levels. While Cys603 is involved in homodimer formation, Cys592 and Cys608 appear to be even more important for the formation of an intramolecular disulfide bond that greatly affects the protein stability as well as plasma membrane targeting of the ABCG2 protein [40,41]. Recent studies have demonstrated that the *N*-linked glycan bound to Asn596 is important for stabilizing nascent ABCG2 proteins by facilitating homodimers in the endoplasmic reticulum (ER) [42,43].

**Figure 5.**
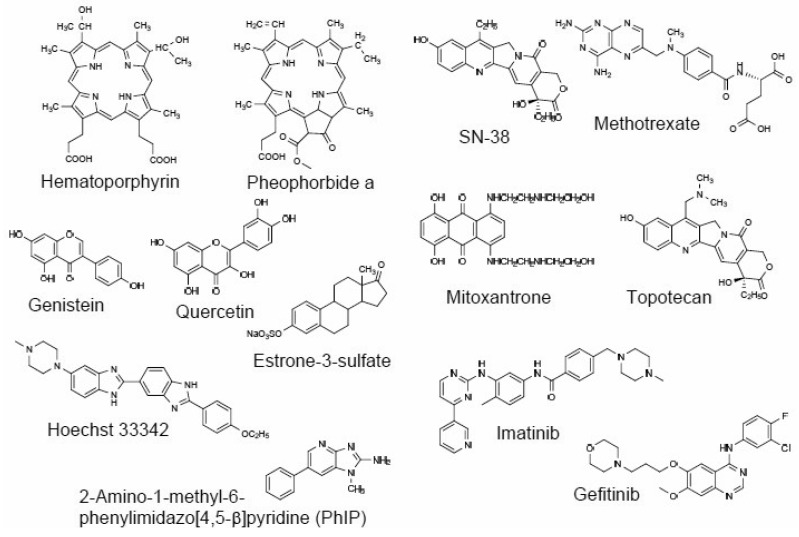
Chemical structures of ABCG2 substrates.

ABCG2 is endogenously expressed in placental trophoblast cells, in the epithelium of the small intestine and liver canalicular membrane, as well as in ducts and lobules of the breast. In particular, the high levels of ABCG2 expression in trophoblast cells suggest that the pump is responsible either for transporting compounds into the fetal blood supply, or for removing toxic metabolites. As demonstrated in Figure 5, ABCG2 transports a variety of drugs, chemicals, and natural compounds. Importantly, ABCG2 substrates include many anticancer drugs, such as methotrexate, mitoxantrone, topotecan, SN-38, imatinib, and gefitinib. The apical localization in the epithelium of the small intestine and colon indicates a possible role of ABCG2 in regulating the uptake of *p.o*. administered drugs [44]. It has recently been reported that gefitinib (iressa) enhanced the oral availability of irinotecan in mice [45], suggestive of the potential inhibition of ABCG2 by gefitinib in the small intestine.

In 2002, the research group of Dr. Schinkel first provided evidence that ABCG2-knockout mice are extremely sensitive to the dietary chlorophyll-breakdown product pheophorbide a, which suggests that ABCG2 expressed in the small intestine plays a critical role in reducing the risk for developing diet-dependent phototoxicity and protoporphyria [46]. Importantly, ABCG2 has a high affinity to porphyrins, e.g., hematoporphyrin and pheophorbide a [47]. ABCG2 transported protoporphyrin, hematoporphyrin, and pheophorbide a in an ATP-dependent manner [48-50]. Since then, the physiological role of ABCG2 has been recognized in terms of porphyrin homeostasis [46], photosensitivity [46-49], PDD [51], and PDT [52,53]. Clinical photosensitizers, such as protoporphyrin, 2-(1-hexyloxethyl)-2-devinyl pyropheophorbide a (photochlor), and benzoporphyrin derivative monoacid ring A (Verteporfin), were transported out of cells by ABCG2, whereas this effect was abrogated by coadministration of imatinib mesylate [54]. By increasing intracellular photosensitizer levels in ABCG2-positive tumors, imatinib mesylate or other ABCG2 transport inhibitors are considered to enhance the efficacy and selectivity of clinical PDD and PDT [54].

## 6. Hypothetical Mechanism Underlying ABCG2-Mediated PpIX Transport

Human ABCG2 exhibits broad substrate specificity toward structurally diverse compounds, as do other ABC transporters, such as ABCB1 (P-glycoprotein/MDR1), ABCC1 (MRP1/GS-X pump), and ABCC2 (MRP2/cMOAT). To gain insight into the relationship between the molecular structure of compounds and the ABCG2-mediated transport activity, we have recently developed a high speed screening method for analyzing the substrate specificity of ABCG2 [47,55].

**Figure 6.**
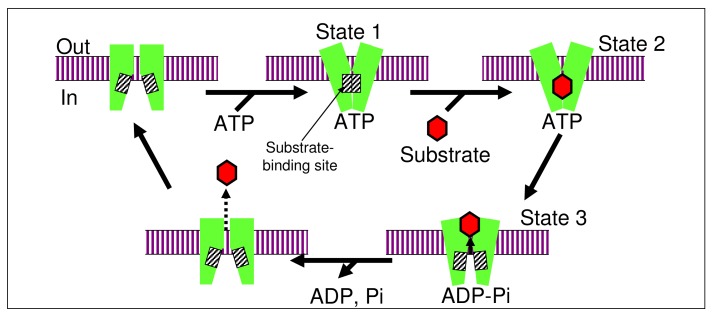
Schematic illustration of ATP-dependent drug transport mediated by ABCG2.

Kinetic data strongly suggest that a substrate molecule is recognized by the ABCG2-ATP complex [56]. Figure 6 illustrates a putative mechanism for ABCG2-mediated transport of a drug across the plasma membrane. According to our hypothesis, the homodimer of ABCG2 first binds two molecules of ATP to form a substrate binding site. A substrate molecule such as gefitinib or PpIX is bound to the substrate binding site of the ABCG2-ATP complex. Hydrolysis of ATP results in a conformational change of the ABCG2 protein, that may push the drug outwards. As ADP and inorganic phosphate (Pi) are released from the ABCG2 protein, the drug is also released from ABCG2 and becomes solvated in the extra cellular milieu or transferred to extracellular partners. With this respect, Desuzinges-Mandon *et al.* [57] have recently shown that the ABCG2 large extracellular loop, ECL3, constitutes a porphyrin-binding domain which is strategically positioned to release the bound porphyrin to extracellular partners. Human serum albumin is suggested to be one of the possible partners [57]. Accurate mechanisms underlying the ABCG2-mediated drug transport across the plasma membrane may be elucidated by X-ray crystallography of the ABCG2 protein taken at different transport stages. However, presently no crystal structures are available for the human ABCG2 protein and protein homology models have not been validated experimentally.

## 7. Inhibition of ABCG2 by Gefitinib, Imatinib, and Cyclin-Dependent Kinase (CDK) Inhibitors

**Figure 7.**
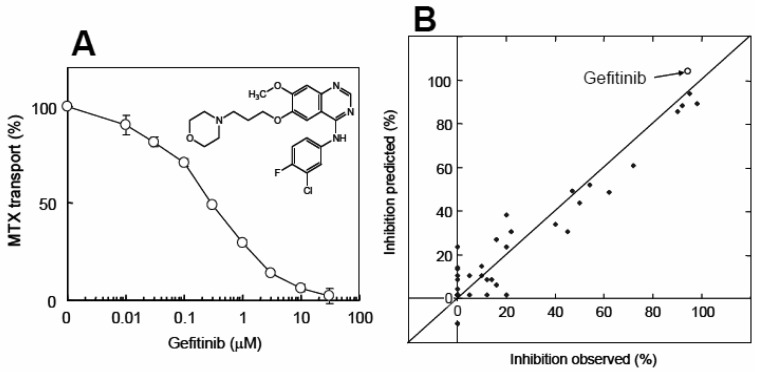
(**A**) Inhibition of ABCG2-mediated MTX transport by gefitinib. Experimental procedures and conditions are described previously [47]. The chemical structure of gefitinib is depicted in this figure. (**B**) Relationship between observed and predicted values in the inhibition of ABCG2-mediated MTX transport by different test compounds. The inhibition by gefitinib is indicated by an open circle (○). Data are from [47].

Protein kinases are potential drug targets for the treatment of a variety of diseases, including cancer [58]. In particular, specific tyrosine kinase inhibitors are rapidly being developed as new drugs for the inhibition of malignant cell growth and metastasis. Most of these newly developed tyrosine kinase inhibitors are hydrophobic, and thus rapidly penetrate the cell membrane to reach intracellular targets. The human genome encodes more than 500 protein kinases, and this protein kinase family has been the subject of intensive research for the development of novel anticancer drugs [59]. Gefitinib, for example, is an anticancer drug that has been developed as an inhibitor for epidermal growth factor receptor (EGFR) tyrosine kinase. Interestingly, gefitinib inhibits ABCG2-mediated transport very effectively. The IC_50_ value was estimated to be 0.28 μM, when methotrexate (MTX) was used as a model substrate (Figure 7A).

To gain insight into the relationship between the chemical structure of test compounds and the inhibition of ABCG2-mediated MTX transport activity, we have performed QSAR analysis by introducing chemical fragmentation codes [47,56]. The *R*^2^ value was estimated to be 0.920 [47]. Statistical significance was determined by *F*-test where the *F* value was calculated to be 50.1 [47]. This *F* value certified the QSAR equation is significant. Based on the QSAR analysis, it is suggested that 10 μM gefitinib inhibits ABCG2-mediated MTX transport almost completely (104% of inhibition) (Figure 7B). As demonstrated in the figure, QSAR analysis-based prediction of transport inhibition was well correlated with the observed values of inhibition.

Recently, another class family of protein kinases has been attracting particular attention. Namely, the cyclin-dependent kinases (CDKs), which regulate critical processes of cell cycle progression and gene transcription essential for cancer cell survival [60]. In cancer, CDKs are deregulated in different ways, such as by the overexpression of cyclin E [61] and loss of p16^INK4A^, a CDK inhibitor [62]. Thus, small-molecule chemicals that specifically regulate or inhibit CDKs are of great interest in drug discovery and development for cancer chemotherapy.

**Figure 8.**
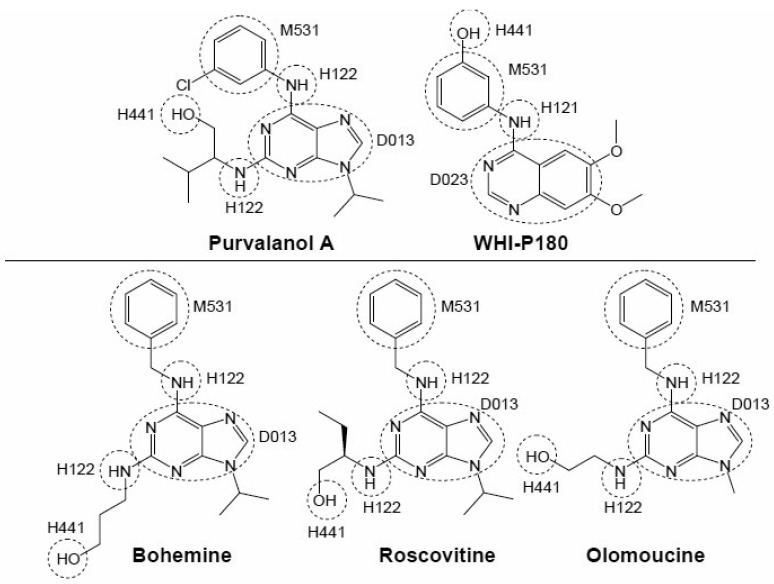
Chemical structures of CDK inhibitors: purvalanol A, WHI-P180, roscovitine, bohemine, and olomoucine. Chemical fragmentation codes involved in these CDK inhibitors were deduced by using the Markush TOPFRAG program (http://thomsonderwent.com/products/patentresarch/markushtopfrag/) (Derwent Information Ltd., London, UK) as described previously [47]. Information of each chemical fragmentation code is briefly summarized in Table 1.

The QSAR analysis revealed that a structure having one amine bonded to one carbon of a heterocyclic ring is an important component for interaction with the ABCG2 protein [47]. In addition, fused heterocyclic ring(s) and two substituents on a carbocyclic ring of the fused heterocyclic ring(s) are also important chemical moieties for the interaction with ABCG2 [47]. Interestingly, many protein kinase inhibitors carry such structural components within their molecules. As shown in Figure 8, the chemical fragmentation codes of purvalanol A, WHI-P180, bohemine, roscovitine, and olomoucine involve H121, H122, D013, D023, and H441 (Table 1) as positive contributors. Based on the QSAR analysis, we hypothesized that those CDK inhibitors would interact with the ABCG2 protein [43,50].

**Figure 9.**
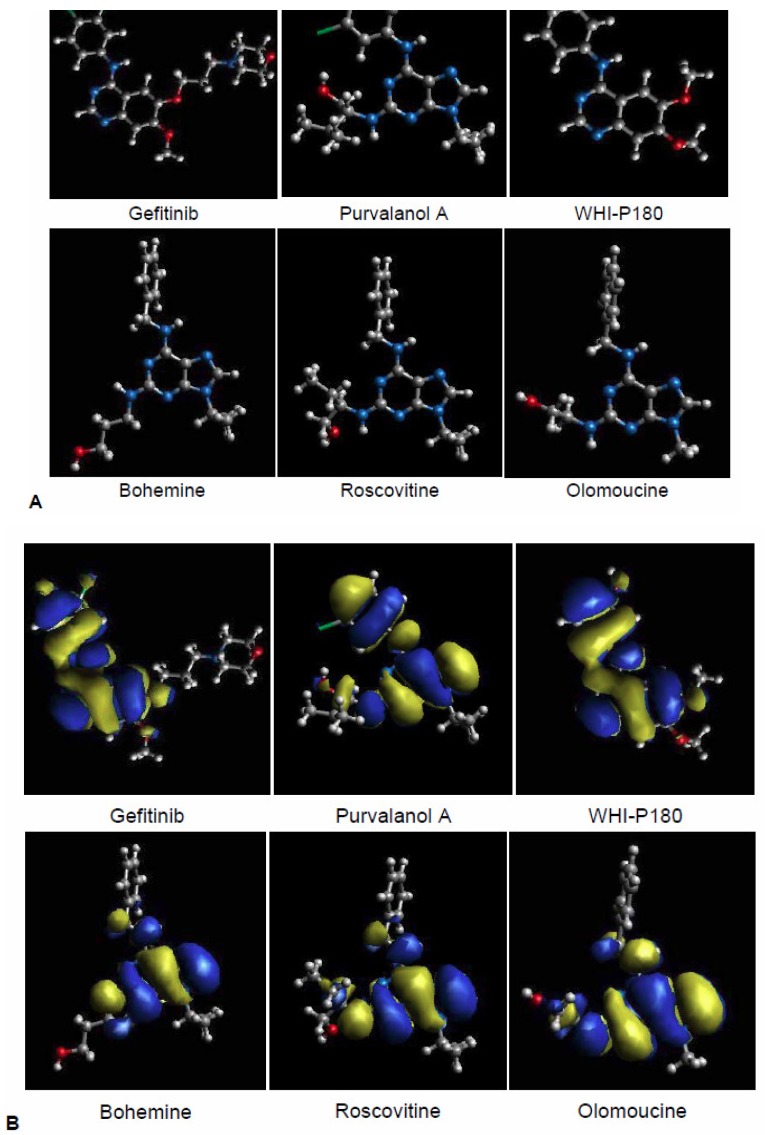
Putative three-dimensional (3D) structures of purvalanol A, WHI-P180, gefitinib, roscovitine, bohemine, roscovitine, and olomoucine. The 3D structures (**A**) and the highest occupied molecular orbits (HOMO) (**B**) of those CDK inhibitors were generated by *ab initio* MO calculation as described previously [50].

To gain further insights into drug-ABCG2 interactions and the three-dimensional (3D) structures of those CDK inhibitors, we performed *ab initio* molecular orbital (MO) calculations based on the restricted Hartree-Fock (RHF) level of theory. The basis set used were MIDI-4, and MO calculations were performed on a work station cluster (NEC Express5800-120Rc-1 geon 2.4GHz x 2) of 32 nodes with the program package AMOSS, which had been developed by NEC (Tokyo, Japan) [50,56]. Figure 9A depicts the 3D structures of purvalanol A, WHI-P180, bohemine, roscovitine, and olomoucine as well as gefitinib. It has become clear that, like gefitinib, purvalanol A and WHI-P180 have a planar structure, whereas bohemine, roscovitine, and olomoucine do not. In the latter CDK inhibitors, the aromatic ring is orthogonal to the purine ring (Figure 9A) [50,56]. As compared with bohemine, roscovitine, and olomoucine, both purvalanol A and WHI-P180 were stronger inhibitors of ABCG2-mediated MTX transport in membrane vesicles. Thus, it is suggested that the planar structure is an important factor for interactions with the active site of ABCG2. Furthermore, the highest occupied molecular orbits (HOMO) of protein kinase inhibitors may play a significant role for stronger interaction with a substrate-binding site(s) of the ABCG2 protein. Indeed, gefitinib, purvalanol, and WHI-P180 have very similar HOMO structures (Figure 9B).

**Figure 10.**
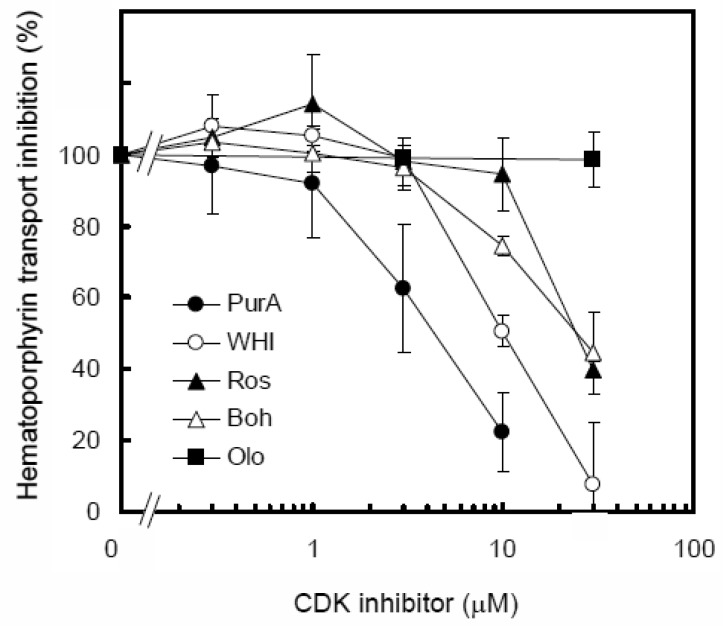
Inhibition of ABCG2-mediated hematoporphyrin transport by purvalanol A, WHI-P180, roscovitine, bohemine, or olomoucine. ABCG2-expressing plasma membrane vesicles (20 μg of protein) were incubated with 20 μM hematoporphyrin in the presence of purvalanol A, WHI-P180, roscovitine, bohemine, or olomoucine (final concentration: 0, 0.3, 1, 3, 10, or 30 μM) in the standard incubation medium (0.25 M sucrose and 10 mM Tris/HEPES, pH 7.4, 1 mM ATP, 10 mM creatine phosphate, 100 μg/mL of creatine kinase, 10 mM MgCl_2_) at 37 °C for 10 min. Hematoporphyrin transported into membrane vesicles was detected [50]. Data are expressed as mean values ± SD (*n* = 5).

Among the CDK inhibitors tested (*i.e.*, purvalanol A, WHI-P180, bohemine, roscovitine, and olomoucine), purvalanol A was found to be the most potent inhibitor for ABCG2-mediated hematoporphyrin transport (Figure 10). Accordingly, it evoked the photosensitivity of ABCG2-expressing Flp-In-293 cells treated with pheophorbide a [50]. Taken together, ABCG2 is considered as one of the critical factors that may affect the efficacy of PDD and PDT of human cancer. It is suggested that the planar structure of inhibitors is an important factor for interactions with the active site of ABCG2.

## 8. Conclusions

Hitherto, many attempts have been made to circumvent ABCG2-mediated multi-drug resistance of human cancer by developing ABCG2-specific inhibitors. Flavonoids, including 6-prenylchrysis and tectochrisin, have been identified as specific inhibitors of ABCG2 [63,64]. In addition, elacridar and tarquidar, ABCB1-inhibitors in clinical trials, are reportedly potent to inhibit human ABCG2 [65,66]. However, long-term administration of an ABCG2 inhibitor can cause drug-induced adverse reactions, such as photo-toxicity [67], and enhance disease risk. Since ABCG2, expressed on the apical side of the proximal tubular cells in human kidney, plays a pivotal role in renal excretion of serum uric acid [68], long-term inhibition of renal ABCG2 can cause gout and/or enhance the risk of cardiovascular disease and diabetes. In this context, a short-termed and limited dose of an ABCG2-specific inhibitor during intra-operative PDD of human brain tumors may be recommended as a practical approach to enhance the efficacy of PDD at border regions of malignant gliomas, and to reduce the risk of drug-induced adverse reactions for cancer patients. To prove this concept, we need to perform further clinical research.

## Figures and Tables

**Table 1.** Descriptors and chemical fragmentation codes (CFC) involved in CDK inhibitors.

**Descriptor**	**CFC**	**Description**
H12		Amine bonded to heterocyclic carbon One Two
	H121	
	H122	
D01		Substituents on a heterocyclic ring of a fused-ring heterocyclic system 2 atoms of a fused heterocyclic ring bear substituents
	D013	
D02		Substituents on a carbocyclic ring of a fused-ring heterocyclic system 2 carbon atoms of a fused carbocyclic ring bear substituents
	D023	
OH		Hydroxy groups
	H401	One -OH group
	H441	One -OH group bonded to aromatic carbon
M531	M531	One carbocyclic system with at least one aromatic ring

## References

[1] Lipson R.L., Baldes E.J. (1960). The photodynamic properties of a particular hematoporphyrin derivative. Arch. Dermatol..

[2] Lipson R.L., Baldes E.J., Olsen A.M. (1961). Hematoporphyrin derivative: a new aid for endoscopic detection of malignant disease. J. Thorac. Cardiovasc. Surg..

[3] Lipson R.L., Baldes E.J., Olsen A.M. (1964). Further evaluation of the use of hematoporphyrin derivative as a new aid for the endoscopic detection of malignant disease. Dis. Chest.

[4] Lipson R.L., Baldes E.J., Gray M.J. (1967). Hematoporphyrin derivative for detection and management of cancer. Cancer.

[5] Gray M.J., Lipson R., Maeck J.V., Romeyn D. (1967). Use of hematoporphyrin derivative in detection and management of cervical cancer. Am. J. Obstet. Gynecol..

[6] Sanderson D.R., Fontana R.S., Lipson R.L., Baldes E.J. (1972). Hematoporphyrin as a diagnostic tool. A preliminary report of new techniques. Cancer.

[7] Dolmans D.E., Fukumura D., Jain R.K. (2003). Photodynamic therapy for cancer. Nat. Rev. Cancer.

[8] Dougherty T.J., Kaufman J.E., Goldfarb A., Weishaupt K.R., Boyle D., Mittelman A. (1978). Photoradiation therapy for the treatment of milignant tumors. Cancer Res..

[9] Stummer W., Novotny A., Stepp H., Goetz C., Reulen H.J. (2000). Fluorescence-guided resection of glioblastoma multiforme by using 5-aminolevulinic acid-induced porphyrins: a prospective study in 52 consecutive patients. J. Neurosurg..

[10] Floeth F.W., Stummer W. (2005). The value of metabolic imaging in diagnosis and resection of cerebral gliomas. Nat. Clin. Pract. Neurol..

[11] Shinoda J., Yano H., Yoshida S., Okumura A., Kaku Y., Iwata T., Sakai N. (2003). Fluorescence-guided resection of glioblastoma multiforme by using high-dose fluorescein sodium. Technical note. J. Neurosurg..

[12] Kajimoto Y., Kuroiwa T., Miyatake S., Ichioka T., Miyashita M., Tanaka H., Tsuji M. (2007). Use of 5-aminolevulinic acid in fluorescence-guided resection of meningioma with high risk of recurrence. Case report. J. Neurosurg..

[13] Lacroix M., Abi-Said D., Fourney D.R., Gokaslan Z.L., Shi W., DeMonte F., Lang F.F., McCutcheon I.E., Hassenbusch S.J., Holland E., Hess K., Michael C., Miller D., Sawaya R. (2001). A multivariate analysis of 416 patients with glioblastoma multiforme: prognosis, extent of resection, and survival. J. Neurosurg..

[14] Hentschel S.J., Sawaya R. (2003). Optimizing outcomes with maximal surgical resection of malignant gliomas. Cancer Control..

[15] Stummer W., Pichmeier U., Meinel T., Wiestler O.D., Zanella F., Reulen H.J. (2006). ALA-Glioma Study Group. Fluorescence-guided surgery with 5-aminolevulinic acid for resection of malignant glioma: a randomized controlled multicenter phase III trial. Lancet Oncol..

[16] Stummer W., Reulen H.J., Vovotny A., Stepp H., Tonn J.C. (2003). Fluorescence-guided resections of malignant gliomas—An Overview. Acta Neurochir. Suppl..

[17] Latunde-Dada G.O., Simpson R.J., McKie A.T. (2006). Recent advances in mammalian haem transport. Trends Biochem. Sci..

[18] Tsiftsoglou A.S., Tsamadou A.I., Papadopoulou L.C. (2006). Heme as key regulator of major mammalian cellular functions: molecular, cellular, and pharmacological aspects. Pharmacol. Ther..

[19] Krishnamurthy P., Xie T., Schuetz J.D. (2007). The role of transporters in cellular heme and porphyrin homeostasis. Pharmacol. Ther..

[20] Wakabayashi K., Tamura A., Saito H., Onishi Y., Ishikawa T. (2006). Human ABC transporter ABCG2 in xenobiotic protection and redox biology. Drug Metab. Rev..

[21] Dubakiene R., Kupriene M. (2006). Scientific problems of photosensitivity. Medicina (Kaunas).

[22] Hindmarsh J.T. (2003). The porphyrias, appropriate test selection. Clin. Chim. Acta.

[23] Norman R.A. (2005). Past and future: porphyria and porphyrins. Skinmed.

[24] Döring F., Walter J., Will J., Föcking M., Boll M., Amasheh S., Clauss W., Daniel H. (1998). Delta-aminolevulinic acid transport by intestinal and renal peptide transporters and its physiological and clinical implications. J. Clin. Invest..

[25] Novotny A., Xiang J., Stummerm W., Teuscher N.S., Smith D.E., Keep R.F. (2000). Mechanisms of 5-aminolevulinic acid uptake at the choroid plexus. J. Neurochem..

[26] Whitaker C.J., Battah S.H., Forsyth M.J., Edwards C., Boyle R.W., Matthews E.K. (2000). Photosensitization of pancreatic tumour cells by delta-aminolaevulinic acid esters. Anticancer Drug Des..

[27] Rodriguez L., Batlle A., Di Venosa G., MacRobert A.J., Battah S., Daniel H., Casas A. (2006). Study of the mechanisms of uptake of 5-aminolevulinic acid derivatives by PEPT1 and PEPT2 transporters as a tool to improve photodynamic therapy of tumours. Int. J. Biochem. Cell Biol..

[28] Kennedy J.C., Pottier R.H. (1992). Endogenous protoporphyrin IX, a clinically useful photosensitizer for PDT. J. Photochem. Photobiol. B: Biol..

[29] Schneckenburger H., König K., Kunzi-Rapp K., Westphal-Frösch C., Rück A. (1993). Time-resolved *in-vivo* fluorescence of photosensitizing porphyrins. J. Photochem. Photobiol. B: Biol..

[30] Weishaupt K.R., Gomer C.J., Dougherty T.J. (1976). Idntification of singlet oxygen as the cytotoxic agent in photoinactivation of murine tumors. Cancer Res..

[31] Pottier R., Truscott T.G. (1986). The photochemistry of hematoporphyrin and related systems. Int. J. Radiat. Biol..

[32] Loh C.S., Vernon D., McRobert A.J., Bedwell J., Bown S.G., Brown S.B. (1993). Endogenous porphyrin distribution induced by 5-aminolevulinic acid in the tissue layer of the gastrointestinal tract. J. Photochem. Photobiol. B: Biol..

[33] Takahashi K., Ikeda N., Nonoguchi N., Kajimoto Y., Miyatake S., Hagiya Y., Ogura S., Nakagawa H., Ishikawa T., Kuroiwa T. (2011). Enhanced expression of coproporphyrinogen oxidase in malignant brain tumors: CPOX expression and 5-ALA-induced fluorescence. Neuro-Oncology.

[34] Hagiya Y., Adachi T., Ogura S., An R., Tamura A., Nakagawa H., Okura I., Mochizuki T., Ishikawa T. (2008). Nrf2-dependent induction of human ABC transporter ABCG2 and hemeoxygenase-1 in HepG2 cells by photoactivation of porphyrins: Biochemical implications for cancer cell response to PDT. J. Exp. Ther. Oncol..

[35] Gupta N., Martin P.M., Miyauchi S., Ananth S., Herdman A.V., Martindale R.G., Podolsky R., Ganapathy V. (2006). Down-regulation of BCRP/ABCG2 in colorectal and cervical cancer. Biochem. Biophys. Res. Commun..

[36] Doyle L.A., Yang W., Abruzzo L.V., Krogmann T., Gao Y., Rishi A.K., Ross D.D. (1998). A multidrug resistance transporter from human MCF-7 breast cancer cells. Proc. Natl. Acad. Sci. USA.

[37] Allikmets R., Schriml L.M., Hutchinson A., Romano-Spica V., Dean M. (1998). A human placenta-specific ATP-binding cassette gene (ABCP) on chromosome 4q22 that is involved in multidrug resistance. Cancer Res..

[38] Miyake K., Mickley L., Litman T., Zhan Z., Robey R., Cristensen B., Brangi M., Greenberger L., Dean M., Fojo T., Bates S.E. (1999). Molecular cloning of cDNAs which are highly overexpressed in mitoxantrone-resistant cells: demonstration of homology to ABC transporter genes. Cancer Res..

[39] Wakabayashi K., Nakagawa H., Adachi T., Kii I., Kobatake E., Kudo A., Ishikawa T. (2006). Identification of cysteine residues critically involved in homodimer formation and protein expression of human ATP-binding cassette transporter ABCG2: a new approach using the flp recombinase system. J. Exp. Ther. Oncol..

[40] Wakabayashi K., Nakagawa H., Tamura A., Koshiba S., Hoshijima K., Komada M., Ishikawa T. (2007). Intramolecular disulfide bond is a critical checkpoint determining degradative fates of ABC transporter ABCG2 protein. J. Biol. Chem..

[41] Wakabayashi-Nakao K., Tamura A., Furukawa T., Nakagawa H., Ishikawa T. (2009). Quality control of human ABCG2 protein in the endoplasmic reticulum: ubiquitination and proteasomal degradation. Adv. Drug Deliv. Rev..

[42] Nakagawa H., Wakabayashi-Nakao K., Tamura A., Toyoda Y., Koshiba S., Ishikawa T. (2009). Disruption of *N*-linked glycosylation enhances ubiquitin-mediated proteasomal degradation of human ATP-binding cassette transporter ABCG2. FEBS J..

[43] Ishikawa T., Nakagawa H. (2009). Human ABC transporter ABCG2 in cancer chemotherapy and pharmacogenomics. J. Exp. Ther. Oncol..

[44] Allen J.D., Schinkel A.H. (2001). Multidrug resistance and pharmacological protection mediated by the breast cancer resistance protein (BCRP/ABCG2). Mol. Cancer Ther..

[45] Stewart C.F., Leggas M., Scheutz J.D., Panetta J.C., Cheshire P.J., Peterson J. (2004). Gefitinib enhances the antitumor activity and oral bioavailability of irinotecan in mice. Cancer Res..

[46] Jonker J.W., Buitelaar M., Wagenaar F., van der Valk M.A., Scheffer G.L., Scheper R.J., Plosch T., Kuipers F., Oude Elferink R.P.J., Rosing H., Beijnen J.H., Schinkel A.H. (2002). The breast cancer resistance protein protects against a major chlorophyll-derived dietary phototoxin and protoporphyria. Proc. Natl. Acad. Sci. USA.

[47] Saito H., Hirano H., Nakagawa H., Fukami T., Oosumi K., Murakami K., Kimura H., Kouchi T., Konomi M., Tao E. (2006). A new strategy of high-speed screening and quantitative structure-activity relationship analysis to evaluate human ATP-binding cassette transporter ABCG2-drug interactions. J. Pharmacol. Exp. Ther..

[48] Tamura A., Watanabe M., Saito H., Nakagawa H., Kamachi T., Okura I., Ishikawa T. (2006). Functional validation of the genetic polymorphisms of human ATP-binding cassette (ABC) transporter ABCG2: identification of alleles that are defective in porphyrin transport. Mol. Pharmacol..

[49] Tamura A., Onishi Y., An R., Koshiba S., Wakabayashi K., Hoshijima K., Priebe W., Yoshida T., Kometani S., Matsubara T., Mikuriya K., Ishikawa T. (2007). *In vitro* evaluation of photosensitivity risk related to genetic polymorphisms of human ABC transporter ABCG2 and inhibition by drugs. Drug Metab. Pharmacokinet..

[50] An R., Hagiya Y., Tamura A., Li S., Saito H., Tokushima D., Ishikawa T. (2009). Cellular phototoxicity evoked through the inhibition of human ABC transporter ABCG2 by cyclin-dependent kinase inhibitors *in vitro*. Pharm. Res..

[51] Ishikawa T., Nakagawa H., Hagiya Y., Nonoguchi N., Miyatake S., Kuroiwa T. (2010). Key role of human ABC transporter ABCG2 in photodynamic therapy and photodynamic diagnosis. Adv. Pharmacol. Sci..

[52] Robey R.W., Steadman K., Polgar O., Bates S.E. (2005). ABCG2-mediated transport of photosensitizers: potential impact on photodynamic therapy. Cancer Biol. Ther..

[53] Busch T.M., Hahn S.M. (2005). Multidrug resistance in photodynamic therapy. Cancer Biol. Ther..

[54] Liu W., Baer M.R., Bowman M.J., Pera P., Zheng X., Morgan J., Pandey R.A., Oseroff A.R. (2007). The tyrosine kinase inhibitor imatinib mesylate enhances the efficacy of PDT by inhibiting ABCG2. Clin. Cancer Res..

[55] Ishikawa T., Sakurai A., Kanamori Y., Nagakura M., Hirano H., Takarada Y., Yamada K., Fukushima K., Kitajima M. (2005). High-speed screening of human ATP-binding cassette transporter function and genetic polymorphisms: new strategies in pharmacogenomics. Methods Enzymol..

[56] Saito H., An R., Hirano H., Ishikawa T. (2010). Emerging new technology: QSAR analysis and MO calculation to characterize the interaction of protein kinase inhibitors with human ABC transporter ABCG2 (BCRP). Drug Metab. Pharmacokinet..

[57] Desuzinges-Mandon E., Arnaud O., Martinez L., Huché F., Di Pietro A., Falson P. (2010). ABCG2 transports and transfers heme to albumin through its large extracellular loop. J. Biol. Chem..

[58] Noble M.E., Endicott J.A., Johnson L.N. (2004). Protein kinase inhibitors: insights into drug design from structure. Science.

[59] Danceyand J., Sausville E.A. (2003). Issues and progress with protein kinase inhibitors for cancer treatment. Nat. Rev. Drug Discov..

[60] Nurse P.M. (2002). Nobel Lecture. Cyclin dependent kinases and cell cycle control. Biosci. Rep..

[61] Porter P.L., Malone K.E., Heagerty P.J., Alexander G.M., Gatti L.A., Firpo E.J., Daling J.R., Roberts J.M. (1997). Expression of cell-cycle regulators p27Kip1 and cyclin E, alone and in combination, correlate with survival in young breast cancer patients. Nat. Med..

[62] Tsihlias J., Kapusta L., Slingerland J. (1990). The prognostic significance of altered cyclin-dependent kinase inhibitors in human cancer. Annu. Rev. Med..

[63] Ahmed-Belkacem A., Pozza A., Muñoz-Martínez F., Bates S.E., Castanys S., Gamarro F., Di Pietro A., Pérez-Victoria J.M. (2005). Flavonoid structure-activity studies identify 6-prenylchrysin and tectochrysin as potent and specific inhibitors of breast cancer resistance protein ABCG2. Cancer Res..

[64] Zhang S., Yang X., Coburn R.A., Morris M.E. (2005). Structure activity relationships and quantitative structure activity relationships for the flavonoid-mediated inhibition of breast cancer resistance protein. Biochem. Pharmacol..

[65] Lagas J.S., van Waterschoot R.A., van Tilburg V.A., Hillebrand M.J., Lankheet N., Rosing H., Beijnen J.H., Schinkel A.H. (2009). Brain accumulation of dasatinib is restricted by P-glycoprotein (ABCB1) and breast cancer resistance protein (ABCG2) and can be enhanced by elacridar treatment. Clin. Cancer Res..

[66] Kannan P., Telu S., Shukla S., Ambudkar S.V., Pike V.W., Halldin C., Gottesman M.M., Innis R., Hall M. (2011). The “specific” P-glycoprotein inhibitor tariquidar is also a substrate and an inhibitor for breast cancer resistance protein (BCRP/ABCG2). ACS Chem. Neurosci..

[67] Tamura A., An R., Hagiya Y., Hoshijima K., Yoshida T., Mikuriya K., Ishikawa T. (2008). Drug-induced phototoxicity evoked by inhibition of human ABC transporter ABCG2: development of *in vitro* high-speed screening systems. Expert Opin. Drug Metab. Toxicol..

[68] Matsuo H., Takada T., Ichida K., Nakamura T., Nakayama A., Ikebuchi Y., Ito K., Kusanagi Y., Chiba T., Tadokoro S. (2009). Common defects of ABCG2, a high-capacity urate exporter, cause gout. A function-based genetic analysis in a Japanese population. Sci. Trans. Med..

